# Protecting Endangered Animal Species

**DOI:** 10.3390/ani14182644

**Published:** 2024-09-12

**Authors:** Chunwang Li

**Affiliations:** 1 Key Laboratory of Animal Ecology and Conservation Biology, Institute of Zoology, Chinese Academy of Sciences, Beijing 100101, China; licw@ioz.ac.cn; 2 College of Life Sciences, University of Chinese Academy of Sciences, Beijing 100049, China

Currently, global biodiversity loss is a growing problem, and more species are endangered and at risk of extinction. Due to the development of human society, the space left for wildlife has become more limited. Therefore, saving endangered species and conserving biodiversity is a matter of urgency. As of 2023, IUCN has assessed 89,856 animal species, 17,416 of which are listed as endangered, accounting for 19.38% of the total animal species assessed [1]. Among them, 11,195 vertebrate species are listed as endangered, accounting for 17.91% of the total vertebrate species assessed. In invertebrates, 6221 species were listed as endangered, accounting for 22.74% of the total invertebrate species assessed. Due to the vulnerability of endangered animals and their sensitivity to environmental degradation and human disturbance, they need to be given more attention. Therefore, the research on conservation of endangered species has naturally become a hotspot in the fields of ecology and conservation biology.

In this Special Issue on Protecting Endangered Species, we collected 17 papers that related to the conservation of endangered species and threatened animals. These publications cover some important aspects of endangered species conservation, such as ecological adaptation of animals, habitat alteration, climate change, population dynamics of endangered animals, conservation translocation, conservation genetics, pollution effect on animals, and construction of protected areas.

Studies have shown that animals adapt to the ambient environment through their physiological regulation and behavioral strategies [2]. Animals with strong ecological adaptability are able to resist uncertain changes in the environment. However, in the context of dramatic changes in the environment, those species that are less adaptable require more attention.

The gut microbiota plays an important role in regulating the physiological function of wild animals [3], while dietary factors and environmental changes could affect the gut microbiota of animals [4]. In the golden snub-nosed monkey (*Rhinopithecus roxellana*), the different fiber intake results in a significant difference in the gut microbiota between the wild and the captive populations [5]. In contrast to wild populations, captive golden snub-nosed monkeys have less beneficial bacteria and more potentially pathogenic bacteria [5]. In captive *Cyprinus chilia*, the gut microbial community structure is significantly changed after release, along with the trend in initially decreasing and then increasing gut fullness [6]. This suggests that the difference in gut microbiota between captive and released animals should be considered in ex situ conservation.

In sympatric animals, the food niche is another issue of concern. Yue et al. (2023) found that there was a significant difference in the food composition of the Tibetan macaque (*Macaca thibetana*) and the gray snub-nosed monkey (*Rhinopithecus brelichi*) [7]. However, the food niches of these two monkey species almost entirely overlapped in winter. Therefore, although the differentiation of dietary habits can reduce niche overlap and interspecific competition, this will be reversed in different seasons.

For animals, habitats are shelters to survive and keep their populations growing. However, many endangered animals are experiencing global changes that lead to habitat degradation or habitat loss, especially climate change [8]. Thus, predicting potential suitable habitats is essential for endangered species, especially in the context of current global climate change.

Among all the vertebrates, amphibians and reptiles are the most vulnerable to climate change. Tao et al. (2024) found that the currently suitable habitats for Wushan salamander (*Liua shihi*) are concentrated in the Daba Mountains, in southwestern China [9]. An optimistic estimate is that under future climate conditions, the area of suitable habitats will increase. Similarly, the main environmental factors influencing the distribution of Szechwan rat snake (*Euprepiophis perlacea*) include the distance from streams and the slope degree, and their potential habitat will not decrease in the context of future climate scenarios [10].

For mammals, climate change also affects the survival of individuals and the habitat of their population. The marbled polecat (*Vormela peregusna*) is a mustelid mammal endemic to Eurasia and listed as a vulnerable (VU) by IUCN due to its low population size and increasing human threats. It is reported that the marbled polecat has a wide range of distribution under current conditions [11]. However, climate change is predicted to severely affect the distribution of the marbled polecat and substantially lead to a significant reduction in the area of suitable habitats in the future [11]. As with terrestrial mammals, the negative effects of climate change are widely found in marine mammals (especially ice-breeding marine mammals). e.g., spotted seals in the North Pacific may face the loss of breeding habitats caused by climate change [12].

In addition to climate change, human activities such as poaching, bycatch, deforestation, overgrazing, urbanization, road killing, and pollution affect endangered animals negatively and directly. It is reported that global extinctions in the marine environment were mainly attributed to overexploitation, followed by invasive species, habitat destruction, trophic cascades, and pollution [13]. Zuo et al. (2023) found that bycatch and stranding incidents occurred widely across the Shandong Peninsula throughout all seasons from 2000 to 2018. Meanwhile, the widespread use of fishing gear was the principal cause of death and injury to finless porpoises during that period [14]. Chilvers and McClelland (2023) reviewed information from pre-emptive captures and translocations of threatened wildlife undertaken during past oil spills and island pest eradications and listed a number of incidents in which these processes have affected animals [15]. They suggested that in order to reduce the negative impact of pollution on endangered animals, wildlife can be captured and transferred before foreseeable contamination occurs [15].

For protecting endangered animals, in situ conservation and ex situ conservation are the two main conservation strategies that are attracting increasing attention, especially in the face of climate change [16]. The most commonly used measure in in situ conservation is the establishment of protected areas. While the methods in ex situ conservation usually include introduction, reintroduction, translocation, and assisted migrations.

The giant panda (*Ailuropoda melanoleuca*) is the flagship species of animal conservation worldwide. In order to effectively protect the giant panda, its habitat, and the entire ecosystem of the giant panda’s range, China has established a number of nature reserves over the past few decades that have developed into the Giant Panda National Park, which covers an area of 27,134 square kilometers. Despite the success of in situ conservation, some small populations of giant pandas are still facing the problems of being non-self-sustaining [17,18]. While the successful conservation of Przewalski’s gazelle (*Procapra przewalskii*) has led to population increase, it has caused them to face another problem, that of excessive density in their current range [19]. To promote the further conservation of these two species, the conservation translocation method based on ecological models and GAP analysis is/will be used to solve the different problems of conservation in the giant panda and Przewalski’s gazelle [17,18,20].

In this Special Issue, some ecological models (e.g., InVEST-HQ model, MaxEnt model, GAP analysis) are reported being used in the study of many endangered species such as Wushan salamander [9], Szechwan rat snake [10], Przewalski’s gazelle [20], spotted seal [12], yellow-throated martens (*Martes flavigula*), and leopard cats (*Prionailurus bengalensis*) [21], to evaluate and predict suitable habitats and potential distribution areas for endangered animals. These ecological models are also used to determine gaps in existing protected areas, identify priority conservation areas [21] or predict potential corridors between habitats [22].

In addition to macro-ecological methods, other methods, such as the conservation genetics method [23,24], remote sensing of biodiversity [25], and passive acoustics techniques [26], have also been used for inventory and monitoring of endangered animals. This suggests that there is a growing number of new technologies being developed and used in the study and the conservation of endangered species, rather than just the traditional ecological methods. Moreover, an expert opinion survey suggests that even in the basic activities of museum-based biological collection, the new scientific methods are relied upon to improve the effectiveness of biocollections for biodiversity conservation [27].

In sum, the contributions cover studies on the ecological adaptation of endangered animals, the effects of climate change and human activities on endangered animals, and the approaches and methods of animal conservation. Although there are fewer than twenty papers and reviews in this Special Issue, they point out some serious problems endangered animals are facing and reflect the research trends in the conservation of endangered species. There is still a long way to go to protect endangered species, and the problems faced by endangered animals need to be addressed on the basis of in-depth research on ecology and conservation biology.
